# ASO Author Reflections: Gastric Cancer Staging: More than Just TNM?

**DOI:** 10.1245/s10434-020-08438-1

**Published:** 2020-04-06

**Authors:** Jakub Chmelo, Alexander W. Phillips

**Affiliations:** Northern Oesophagogastric Unit, Newcastle upon Tyne, UK

## Past

Pre-operative clinical staging of gastric cancer patients is currently based on TNM classification.^1^ The disease stage may influence patient management and provide important prognostic information to help counsel patients. The importance of additional information from the cancer specimen regarding lymphatic, venous or perineural invasion in the context of neo-adjuvant chemotherapy for gastric adenocarcinoma remains unclear. These have been identified as important within other cancer groups but not necessarily incorporated into the staging of the disease.^2^ The question remains, are these factors associated with poorer outcomes in the post-neoadjuvant gastric cancer cohort? Past studies have evaluated their impact in a neoadjuvant naive group.^3^^,^^4^ Most importantly, could knowledge about these factors influence our decision-making about treatment strategies of this aggressive disease?

## Present

This study reveals additional prognostic factors for patients with gastric adenocarcinoma who were treated with neo-adjuvant chemotherapy and surgery.^5^ The presence of lymphatic, venous and perineural invasion are features of aggressive tumours. These histological findings in the resection sample may play a prognostic role. Further, they could potentially help stratify patients at higher risk of recurrence and aid the decision-making process with regards to administration of adjuvant chemotherapy. This may be particularly useful for patients who are node negative. Knowledge about the presence of these factors preoperatively [endoscopic mucosal resection (EMR) or biopsy based] might also lead to different treatment strategies. This could be the case especially for patients who are staged as having node-negative disease, where an earlier ‘T-stage’ may preclude them from neoadjuvant treatment.

## Future

Future research studies assessing various adjuvant strategies in gastric cancer patients should incorporate information about these prognostic factors. Data need to be collected to establish their full prognostic significance. As a better understanding is developed, the prognostic significance of these histopathological factors may influence decision-making of multidisciplinary teams involved in looking after gastric cancer patients. Surgery and lymphadenectomy continue to be the key components for treating gastric cancer patients. However, the future lies in tailoring treatment specifically to patients and their tumour biology to minimise unnecessary interventions and their side-effects and ensure an optimum therapy.
